# Abdominal aortic spectral Doppler combined with echocardiography can improve the diagnostic sensitivity of aortic coarctation in pediatric patients

**DOI:** 10.3389/fped.2025.1541643

**Published:** 2025-07-15

**Authors:** Qianjun Liu, Yuan Hu, Yinghui Peng, Wenfeng Li, Wenjuan Chen, Jinwen Luo, Jinqiao Liu, Xicheng Deng

**Affiliations:** ^1^ Department of Ultrasound, The Affiliated Children’s Hospital of Xiangya School of Medicine, Central South University (Hunan Children’s Hospital), Changsha, China; ^2^ Department of Cardiothoracic Surgery, The Affiliated Children’s Hospital of Xiangya School of Medicine, Central South University (Hunan Children’s Hospital), Changsha, China

**Keywords:** pediatric patients, coarctation of the aorta, abdominal aortic spectral Doppler, echocardiography, two-dimensional

## Abstract

**Objectives:**

This study aimed to investigate the clinical value of abdominal aortic spectral Doppler combined with echocardiography in the diagnosis of aortic coarctation in pediatric patients.

**Methods:**

Pediatric patients with aortic coarctation, diagnosed by computed tomography angiography (CTA) and surgically confirmed, were retrospectively enrolled. These patients were divided into two groups based on the availability of abdominal aortic spectral Doppler. Additionally, both abdominal aortic spectral Doppler and echocardiographic data were collected for the normal group. All data were compared and analyzed to determine the reasons for discrepancies in diagnostic results.

**Results:**

No significant differences were observed in baseline characteristics among the three groups (*p* > 0.05). There were statistically significant differences in aortic isthmus velocity and aortic isthmus *Z*-scores between the normal group and the two patient groups (*p* < 0.05), but there were no significant differences in aortic isthmus velocity or aortic isthmus *Z*-scores between the two patient groups (*p* > 0.05). The abdominal aortic spectral Doppler group demonstrated significantly decreased peak systolic velocity (PSV), prolonged acceleration time (AT), and reduced pulsatility index (PI) and resistance index (RI) compared with controls (*p* < 0.05). Echocardiographic detection rates differed between groups: non-abdominal aortic spectral Doppler group, 59 true-positive coarctation cases (sensitivity 85.5%, false-negative rate 14.5%); abdominal aortic spectral Doppler group, 75 true-positive cases (sensitivity 96.2%, false-negative rate 3.8%). The combined diagnostic model incorporating abdominal aortic PSV, AT, and aortic isthmus *Z*-score achieved superior performance (AUC = 0.98), significantly outperforming individual parameters.

**Conclusions:**

Abdominal aortic spectral Doppler combined with echocardiography can improve the diagnostic sensitivity of aortic coarctation in pediatric patients and can be used as an important indirect imaging approach in clinical practice to reduce missed diagnoses of aortic coarctation.

## Introduction

Aortic coarctation refers to localized narrowing of the aortic lumen, occurring in the isthmus (the junction between the origin of the left subclavian artery and the end of the aortic arch at the ductus arteriosus) in 95% of cases (1). Transthoracic echocardiography is the preferred screening method for heart disease in pediatric patients. Prompt diagnosis and surgical treatment can significantly increase the quality of life, growth, and development of affected children and decrease long-term mortality rates. However, suprasternal notch ultrasound imaging sometimes yields suboptimal quality. When the coarctation involves the distal descending aorta, visualization may be inadequate, leading to reduced diagnostic accuracy for aortic coarctation in older pediatric patients and adults. During aortic stenosis, the perfusion pressure below the narrowed area is diminished. Owing to the good compliance of the arteries distal to the stenosis, there is a significant decrease in the systolic peak and prolongation of the acceleration time (AT) when the blood flow velocity is measured with ultrasound, a phenomenon referred to as the “tardus‒parvus waveform.” Initially, the tardus–parvus waveform was used to detect reduced flow velocity and perfusion in the distal renal artery secondary to main renal artery stenosis (2). Adult studies have demonstrated that assessing blood flow changes in the abdominal aorta and renal arteries can improve the detection rate of aortic coarctation (3, 4). Abdominal aortic flow alterations have also been investigated as hemodynamic predictors of aneurysm rupture risk (5–7) and for monitoring disease activity in vasculitides such as Takayasu arteritis (8). However, most existing research has focused on adult populations, with relatively limited data available for pediatric patients. When aortic coarctation causes hemodynamic abnormalities in vessels below the affected site, we can infer the developmental state of the aorta through abdominal aortic spectral Doppler, which can increase the vigilance of doctors regarding aortic arch coarctation, especially when a clear image cannot be obtained from the suprasternal notch. Consequently, this study aimed to investigate whether the combination of abdominal aortic spectral Doppler with echocardiography could enhance diagnostic sensitivity for aortic coarctation in pediatric patients.

## Materials and methods

### Study data and population

We retrospectively collected data from pediatric patients with aortic coarctation diagnosed using computed tomography angiography (CTA) and confirmed during surgery in our hospital from January 2018 to January 2024. The enrolled pediatric patients were required to have undergone both echocardiography and CT angiography (CTA) examinations. Patients who underwent CTA but not echocardiography or whose indicator data were missing or incomplete were excluded. The collected data included height, weight, age, aortic isthmus diameter, aortic isthmus velocity, abdominal aorta peak systolic velocity (PSV), acceleration time (AT), pulsation index (PI), resistance index (RI), and the aortic isthmus Z-score. The pediatric patients were divided into two groups based on whether abdominal aortic spectral Doppler was obtained. One group was the non-abdominal aortic spectral Doppler group (in the early stage, abdominal aortic spectral Doppler was not included in the routine measurement of echocardiography because of insufficient understanding of abdominal aortic spectral Doppler), and the other group was the abdominal aortic spectral group. Additionally, we included age-matched children without cardiac disease who underwent CTA during the same period due to a diagnosis of lung diseases, serving as controls for the abdominal aortic spectral Doppler group. A total of 389 patients underwent CTA for lung disease, of whom 145 patients underwent echocardiography. There were 100 children among the 145 cases who were evaluated with abdominal aortic spectral Doppler (they were finally included in the study). Our institution has standardized the integration of abdominal aortic spectral Doppler assessment into all routine echocardiographic examinations, regardless of whether congenital heart disease was suspected. The data on abdominal aortic spectral Doppler in the normal group were also obtained in this way. This imaging confirmed normal aortic arch development in these patients. A total of 289 cases were excluded due to missing data. The study was approved by all relevant committees, and every patient provided written informed consent. All the methods were performed in accordance with relevant regulations.

### Instruments and methods

#### Sedation

For uncooperative pediatric patients, sleep was restricted for 4 h prior to the examination. Ten minutes before the examination, the parents were encouraged to soothe the child to sleep. If a pediatric patient could not fall asleep naturally during the examination, a rectal infusion of 10% chloral hydrate at a dose of 0.5  ml/kg was administered to induce deep sleep, and scanning was conducted while the child was in a calm breathing state.

#### Ultrasound

**Figure 1:**
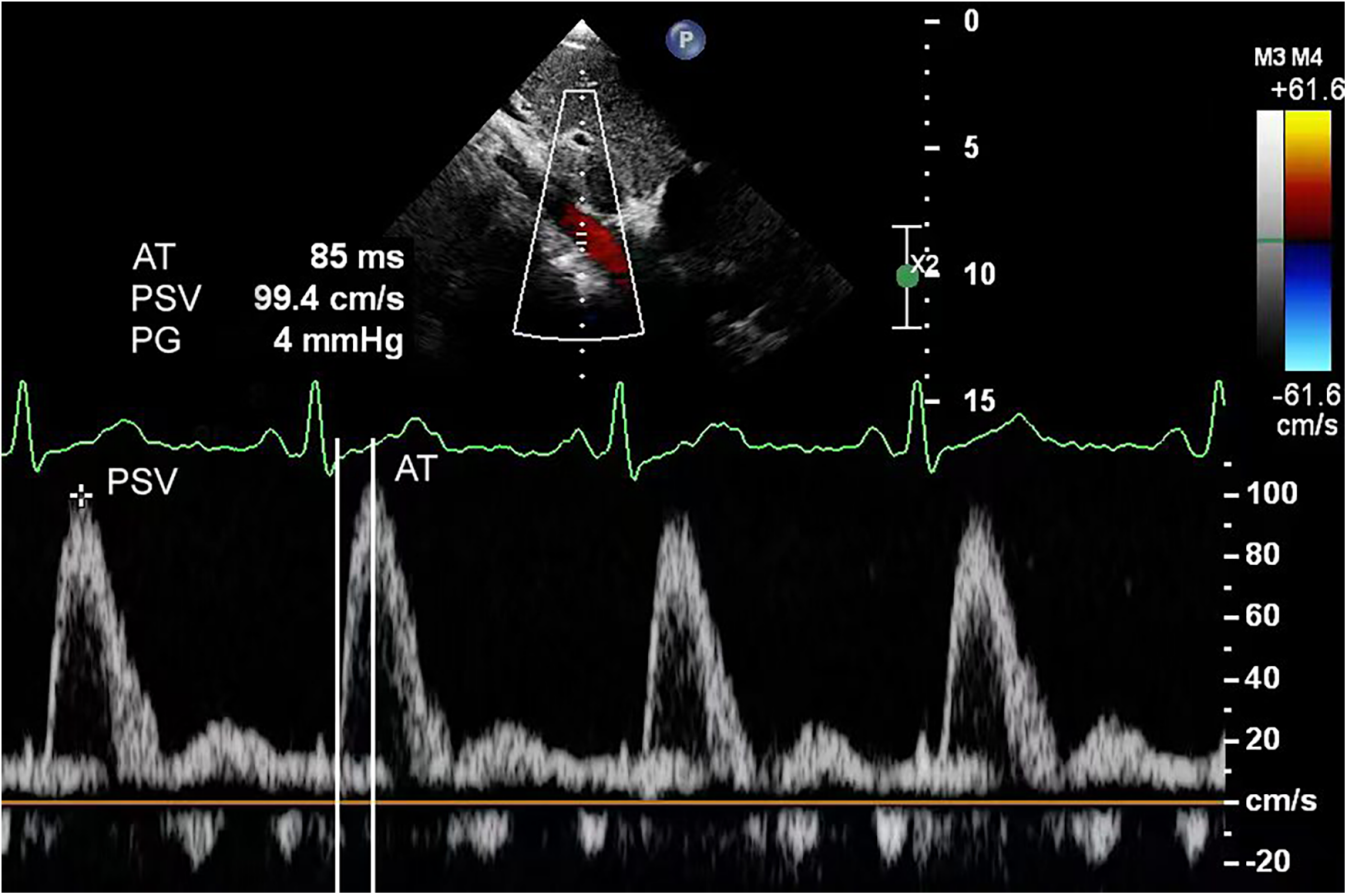
Abdominal aortic spectral Doppler in a 2-month-old infant in the normal group.

**Figure 2:**
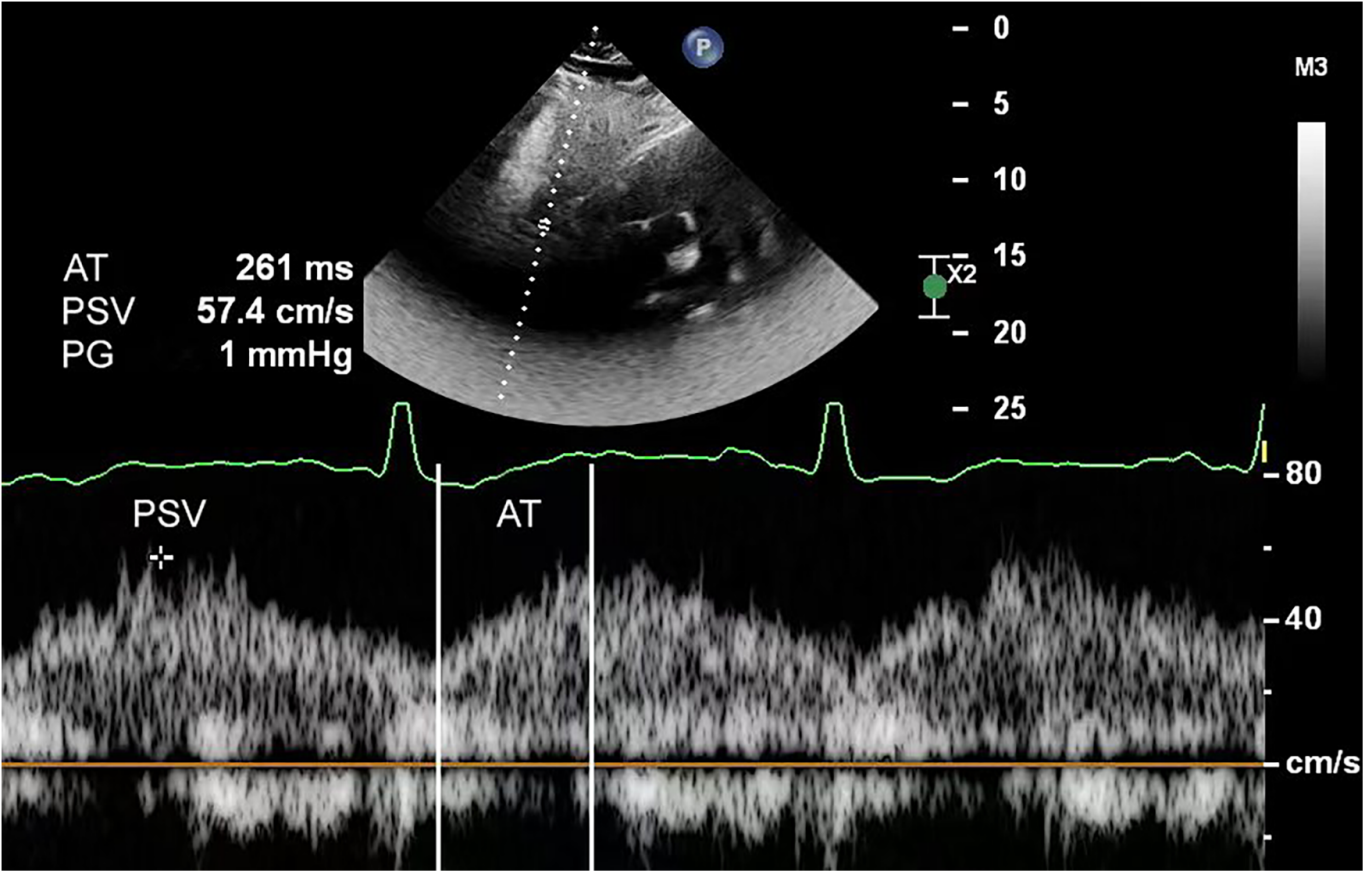
Abdominal aortic spectral Doppler in a 1-month-old infant with aortic coarctation showed a typical tardus–parvus waveform. The echocardiogram's suprasternal notch view did not show aortic coarctation due to interference.

For echocardiography, we used a Philips EPIQ 7C color Doppler ultrasound system equipped with S5-1 (frequency range, 1–5 MHz) and S8-3 (frequency range, 3–8 MHz) phased array probes. Pediatric patients were examined in the supine or left lateral decubitus position, following standard scanning planes (parasternal, apical, subcostal, and suprasternal notch) as recommended by the American Society of Echocardiography guidelines (9). These views were utilized to evaluate cardiac structural abnormalities, including but not limited to ventricular septal defects, atrial septal defects, and aortic valve stenosis. In the abdominal aortic spectral Doppler group, measurements were obtained at the diaphragmatic level using the subcostal view. The following parameters were recorded: aortic isthmus diameter, aortic isthmus flow velocity, abdominal aortic peak systolic velocity (PSV), acceleration time (AT), pulsatility index (PI), and resistance index (RI). The aortic isthmus was measured between the left subclavian artery and the ductus arteriosus. In cases with ductus arteriosus closure, measurements were limited to within 2 cm distal to the origin of the left subclavian artery's posterior margin. The aortic isthmus *Z*-score was calculated using a previously reported formula (9, 10). Peak velocity at the aortic isthmus was measured at maximal systole using spectral Doppler ultrasonography. The spectral Doppler waveform of a normal abdominal aorta demonstrates a triphasic pattern: (1) a sharply ascending, narrow, positive systolic peak; (2) a small negative wave with relatively low flow velocity in early diastole; and (3) a positive low-velocity flow wave during mid-to-late diastole (Figure 1). Abnormal flow spectra were defined as waveforms showing reduced systolic upslope velocity, delayed diastolic deceleration, and loss of the normal triphasic pattern (Figure 2). The PSV in the abdominal aorta was measured at maximum systole. AT was measured from the starting point of the systolic spectrum to the peak of the early systolic wave or the point where the early systolic wave disappeared. However, if these features could not be identified, the AT was measured at the highest point of the spectrum. Throughout measurements, the Doppler beam angle was maintained at ≤20°relative to the direction of blood flow. Written informed consent was obtained from the individual(s), and minor(s)' legal guardian/next of kin, for the publication of any potentially identifiable data included in this article.

#### CTA

**Figure 3:**
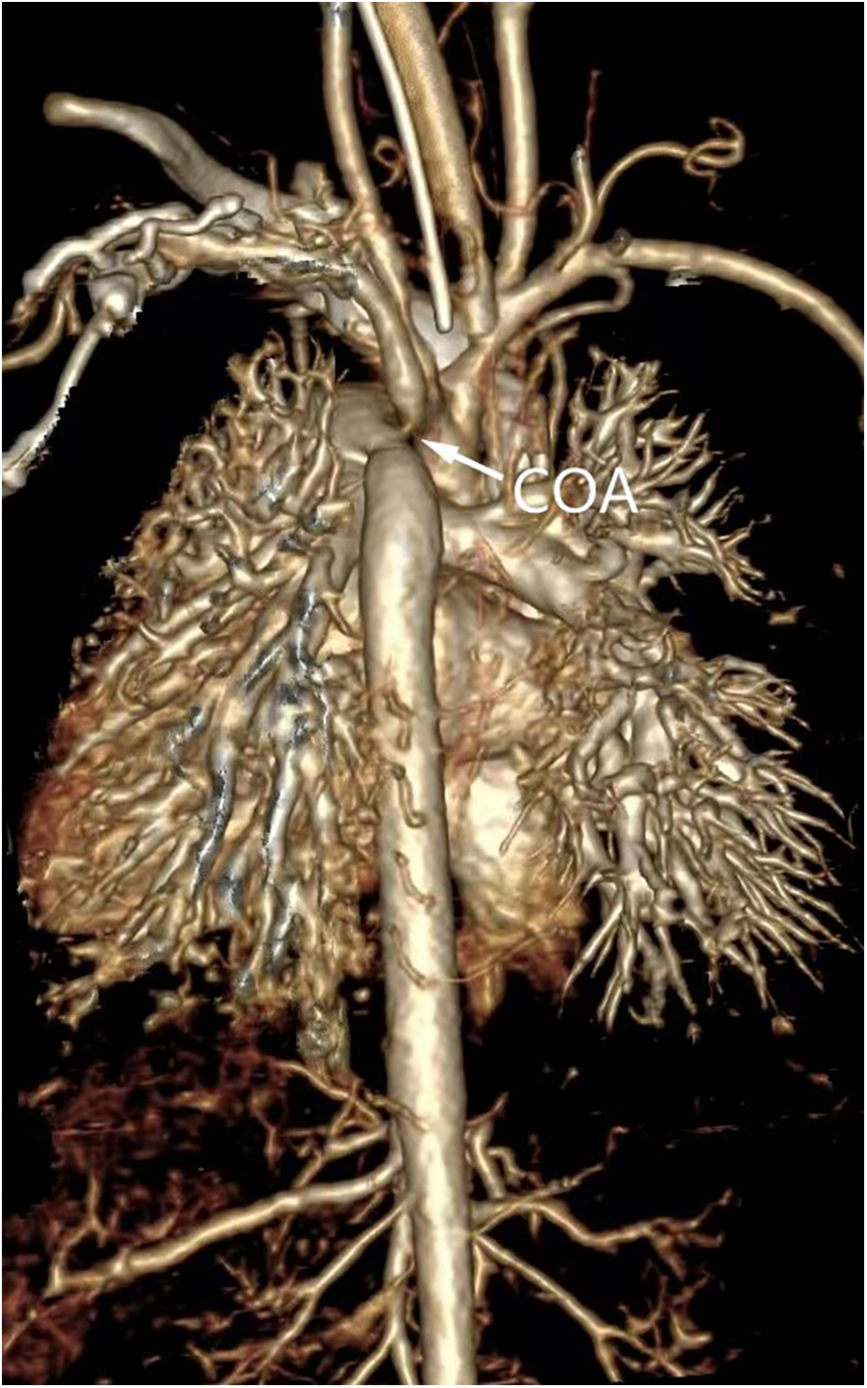
Three-dimensional volumetric imaging of a 1-month-old infant for diagnosis of aortic coarctation by computed tomography angiography.

**Figure 4:**
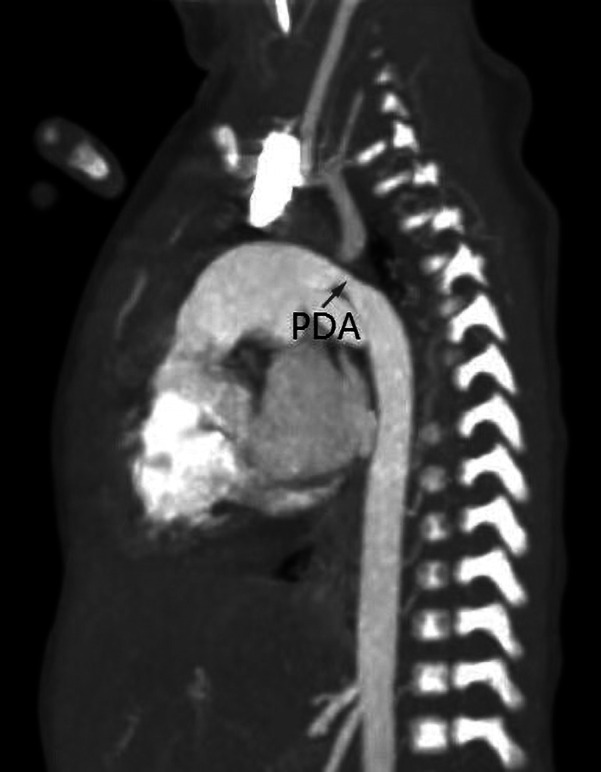
Descending aortic coarctation by computed tomography angiography in a 1-month-old infant. A large patent ductus arteriosus may serve as a source of blood flow to the descending aorta.

Physical examination revealed abnormal heart murmurs, and ultrasound examination revealed aortic arch stenosis. CTA examination was then performed for diagnosis (Figure 3, 4). For children with unexplained abnormal blood pressure, blood pressure differences, short stature that did not match the results of echocardiography, poor echocardiographic windows, and certain thoracic diseases (such as tumors and pulmonary sequestration), cardiac CTA was performed. A GE Revolution 256-slice spiral CT scanner was used, and the patients' genitalia were shielded with lead strips prior to scanning. The scanning range extended from the lung apices to the diaphragm. The non-ionic contrast agent iohexol (320 mgI/ml) was used at a dose of 2–3 ml/kg of body weight. The injection rate ranged from 1.0 to 2.0 ml/s. The scan parameters included a tube voltage of 80–100 kV, a tube current of 80–120 mAs, and a slice thickness of 0.3 mm. According to the positional relationship between the narrowing site and the ductus arteriosus, aortic coarctation was divided into the preductal type and the postductal type.

### Statistical analyses

Statistical analysis was performed using SPSS version 27.0 software. For count data, a normality test was conducted. Data conforming to a normal distribution are expressed as mean ± standard deviation, while non-normally distributed metric data are expressed as median (interquartile range) [M (Q1–Q3)]. For comparisons between two independent samples, normally distributed continuous variables were analyzed using the independent samples *t*-test, and non-normally distributed variables were analyzed using the non-parametric rank sum test. Analysis of variance was used for comparisons among multiple groups. Count data are presented as *n* (%), and intergroup comparisons were performed using the chi-square (*χ*^2^) test. Receiver operating characteristic (ROC) curves were plotted, and the area under the curve (AUC) was calculated to assess the efficiency of the different parameters in diagnosing aortic coarctation. Multivariate analysis was performed using logistic regression. *p* < 0.05 was considered to indicate statistical significance.

## Results

### General information

Our study included 147 pediatric patients diagnosed with aortic coarctation (92 males and 55 females), with ages ranging from 1 day to 15 years (median: 2 months). The control group consisted of 100 subjects (54 males, 46 females) aged 1 day to 16 years (median, 2 months). A total of 78 patients were assigned to the abdominal aortic spectral Doppler group, while 69 comprised the non-abdominal aortic spectral Doppler group. The cohort included 102 preductal and 45 postductal aortic coarctation cases. The anatomical classifications were as follows: isolated coarctation (*n* = 27), coarctation with ventricular septal defect (VSD) (*n* = 58), coarctation with atrial septal defect (ASD) (*n* = 23), coarctation with both VSD and ASD (*n* = 29), hypoplastic aortic arch (*n* = 8), and aortic valve stenosis (*n* = 2). Among the 147 pediatric patients who underwent surgical treatment, there were seven total deaths (4.7%), including four cases (2.7%) from pulmonary hypertensive crisis and three cases (2.0%) from respiratory distress syndrome (RDS). The remaining 140 patients (95.2%) were successfully discharged.

There were no statistically significant differences among the three groups in terms of baseline characteristics (*p* > 0.05) (Table 1). There were statistically significant differences in aortic isthmus velocity and aortic isthmus *Z*-scores between the normal group and the two patient groups (*p* < 0.05), but there were no significant differences in aortic isthmus velocity or aortic isthmus *Z*-scores between the two patient groups(*p* > 0.05) (Table 1). The abdominal aortic spectral Doppler group demonstrated significantly decreased peak systolic velocity (PSV), prolonged acceleration time (AT), and reduced pulsatility index (PI) and resistance index (RI) compared with controls (*p* < 0.05) (Table 1). Echocardiographic detection rates differed between groups: non-abdominal aortic spectral Doppler group, 59 true-positive coarctation cases (sensitivity 85.5%, false-negative rate 14.5%); abdominal aortic spectral Doppler group, 75 true-positive cases (sensitivity 96.2%, false-negative rate 3.8%). The sensitivity of abdominal aortic spectral Doppler combined with echocardiography for the diagnosis of aortic arch constriction was greater than that of echocardiography alone (*p* < 0.05) (Table 2).

**Table 1:** Comparison of data among groups.

Group	Normal group (*N* = 100)	Abdominal aortic spectral group (*N* = 78)	Non-abdominal aortic spectral group (*N* = 69)	*p*
Age (m）	2 (1.6–11)	2.5 (1.3–10.6)	3 (1.5–10.5)	0.272
Height(cm)	58 (55–70)	56 (55–68.5)	58 (56–68)	0.543
Weight (kg)	5.3 (4.1–7.4)	4.9 (4.3–7.5)	5.2 (4.2–7.2)	0.876
Aortic isthmus *Z*-score	−0.58 ± 2	−8.01 ± 2.45*	−8.28 ± 2.61	<0.001
Aortic isthmus velocity (cm/s)	133.27 ± 8.0	278.9 ± 87.3*	262.4 ± 70.1	<0.001
PSV (cm/s)	111.26 ± 24.6	35.10 ± 7.0	/	<0.05
AT (ms)	74.66 ± 6.9	175.95 ± 19.97	/	<0.05
PI	2.41 ± 0.98	0.63 ± 0.17	/	<0.05
RI	0.91 ± 0.09	0.41 ± 0.19	/	<0.05

Data were non-normally distributed metric data and presented as median (interquartile range) [M (Q1–Q3)]. Normally distributed data were presented as mean ± standard deviation (SD).

PSV, peak systolic velocity; AT, acceleration time; PI, pulsatility index; RI, resistive index.

* There were no statistically significant difference in aortic isthmus velocity or aortic isthmus *Z*-score between the abdominal aortic spectral group and the non-abdominal aortic spectral group.

**Table 2:** Diagnostic sensitivity of combined abdominal aortic spectral Doppler and echocardiography for aortic coarctation.

Group	Number of cases diagnosed by echocardiography (*N*)	Number of missed cases by echocardiography( *N*)	Diagnostic sensitivity (%)
Non-abdominal aortic spectral group (*N* = 69)	59	10	85.5%
Abdominal aortic spectral group (*N* = 78)	75	3	96.2%
*χ* ^2^	-	-	5.148
*P*	-	-	0.023

### ROC curve analysis of the value of the abdominal aortic PSV, AT, and aortic isthmus *Z*-score for diagnosing aortic coarctation

**Figure 5:**
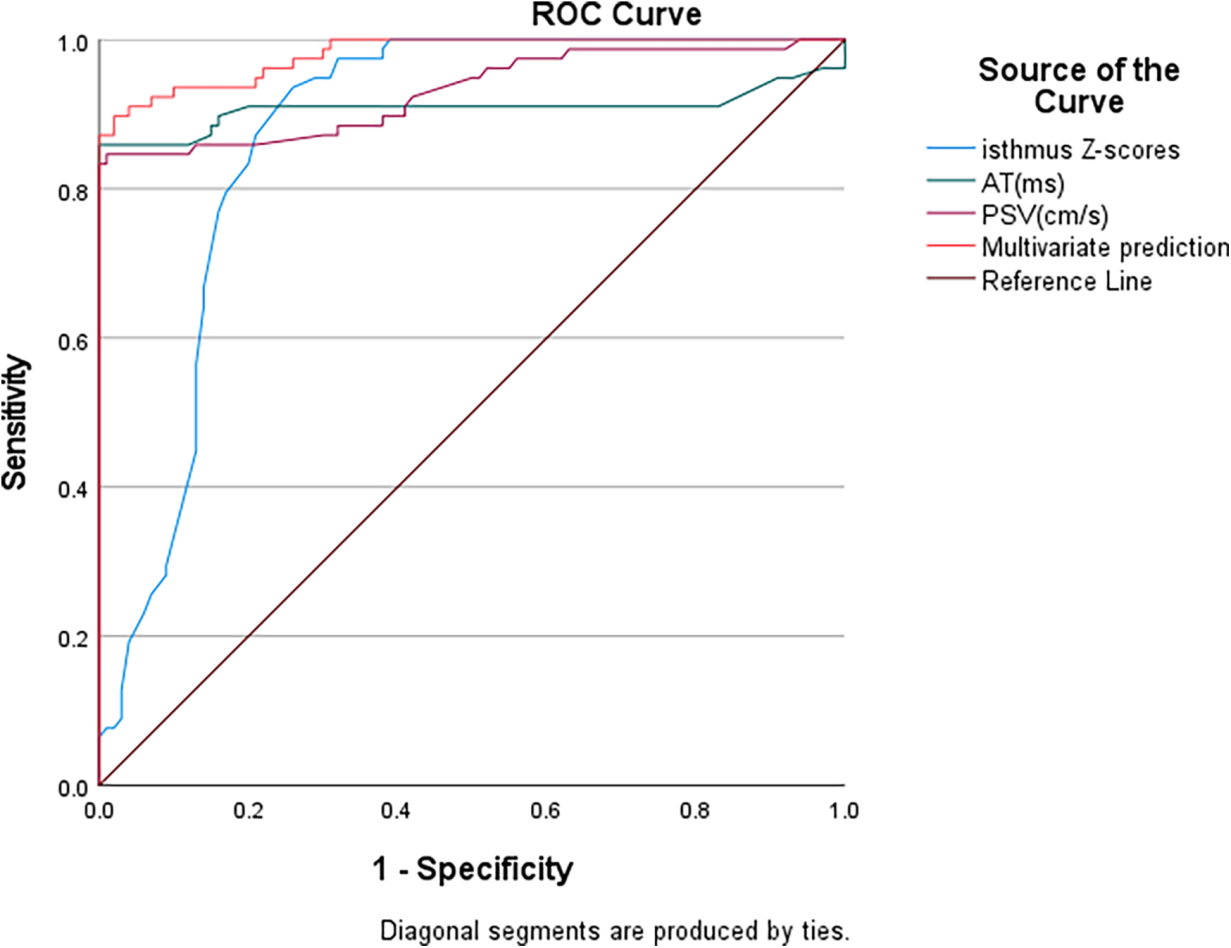
ROC curve analysis of aortic coarctation. PSV, peak systolic velocity; AT, acceleration time.

The area under the curve (AUC) values for diagnosing aortic coarctation were 0.930 for abdominal aortic peak systolic velocity (PSV), 0.908 for acceleration time (AT), and 0.87 for aortic isthmus *Z*-score. When combining abdominal aortic PSV, AT, and aortic isthmus *Z*-score for diagnosis, the AUC increased to 0.98 (Figure 5), demonstrating that the combination of these parameters had the greatest diagnostic value. Using the Youden index, we established the following optimal cutoff values: abdominal aortic PSV, 43.5 cm/s (sensitivity 83.3%, specificity 100%); abdominal aortic systolic AT, 153.5 ms (sensitivity 85.9%, specificity 100%); aortic isthmus *Z*-score, −5.1 (sensitivity 97%, specificity 68%).

## Discussion

Aortic coarctation is a congenital heart disease, and the mechanism by which it occurs during embryonic development remains unclear. Studies suggest that it is associated with various factors, such as abnormalities in arterial duct tissue, hemodynamic alterations, abnormal neural crest development, genetic factors, and gene mutations (11). The incidence rate is approximately 0.03%, and it accounts for 5%–7% of all congenital heart diseases (12). It is more common in males than in females, with most patients being diagnosed within 1–6 months after birth. Cases of mild or moderate stenosis that remain undiagnosed beyond infancy are not uncommon (13, 14). Adolescents or adults may have a history of exercise intolerance or hypertension, occasionally presenting with specific complaints of lower limb fatigue. Severe stenosis may even remain asymptomatic when collateral circulation is well established.

The “tardus–parvus waveform” has been widely used in diagnosing stenotic lesions of the abdomen and peripheral blood vessels (15, 16), especially during evaluation and follow-up after kidney or liver transplantation (17–19). Our research group believes that the causes of changes in the renal artery tardus–parvus waveform are diverse. In addition to aortic coarctation, severe stenosis of the abdominal aorta at the level of the diaphragm to above the origin of the bilateral renal arteries can also lead to changes in the renal artery tardus‒parvus waveform. Furthermore, a change in the tardus–parvus waveform itself is also an indirect diagnostic indicator. When indirect signs are present, the possible causes should be narrowed as much as possible. We believe that using the tardus‒parvus waveform of the abdominal aorta at the level of the diaphragm as an indirect diagnostic indicator of aortic coarctation is a reasonable approach. Therefore, when we detect the presence of a tardus–parvus waveform in the abdominal aorta, we can infer the presence of stenosis in the vessels above it, regardless of whether the view of the aortic arch from the suprasternal notch is clear. Thus, we conducted this study to verify the reliability of abdominal aortic spectral Doppler for diagnosing aortic coarctation. Our analysis revealed that conventional transthoracic echocardiography alone demonstrated an 85.5% diagnostic sensitivity for aortic coarctation. However, the combination of abdominal aortic spectral Doppler and echocardiography had a sensitivity of 96.2% for the diagnosis of aortic coarctation. The area under the curve (AUC) values for diagnosing aortic coarctation were 0.930 for abdominal aortic peak systolic velocity (PSV), 0.908 for acceleration time (AT), and 0.87 for aortic isthmus *Z*-score. When combining abdominal aortic PSV, AT, and aortic isthmus *Z*-score for diagnosis, the AUC increased to 0.98, demonstrating that the combination of these parameters had the greatest diagnostic value. Using the Youden index, a cutoff value of 43.5 cm/s for the abdominal aortic PSV yielded a sensitivity of 83.3%, and the specificity was 100%. Similarly, a cutoff value of 153.5 ms for the abdominal aortic systolic AT yielded a sensitivity of 85.9%, and the specificity was 100%. A cutoff value of −5.1 for the aortic isthmus *Z*-score yielded a sensitivity of 97%, and the specificity was 68%. The majority of patients were infants with adequate windows for aortic isthmus visualization. However, the diagnosis of aortic coarctation was missed in seven patients above 8 years of age. The sonographer may have missed the diagnosis of aortic coarctation because of unsatisfactory scanning from the suprasternal notch or failure to conduct a standardized, meticulous examination. In three newborns, stenosis below the aortic isthmus was not clearly visible due to interference, leading to missed diagnoses. In three patients where combined abdominal aortic spectral Doppler and echocardiography failed to detect aortic coarctation, subsequent analysis identified significant hemodynamic confounding factors: large patent ductus arteriosus (PDA) and well-developed collateral circulation to the lower extremities. These compensatory mechanisms masked the characteristic tardus–parvus waveform in abdominal aortic flow spectra. Five patients exhibited normal aortic isthmus morphology on suprasternal notch imaging, yet abdominal aortic spectral Doppler demonstrated characteristic tardus–parvus waveforms. Based on these hemodynamic abnormalities, we provisionally diagnosed aortic coarctation, which subsequent CTA confirmed as thoracic aortic segmental stenosis. A decrease in the PSV and a prolonged AT on abdominal aortic spectral Doppler suggest significant stenosis of upstream vessels, leading to a marked reduction in perfusion pressure and flow in the downstream stenotic vessels. In our study, 13 pediatric cases of aortic coarctation initially missed by echocardiography were ultimately diagnosed by CT angiography (CTA). Due to observed discordance between their clinical presentations (notably unexplained hypertension and growth retardation) and initial echocardiographic results, repeat imaging confirmation was required. While the diagnostic accuracy of abdominal aortic spectral Doppler may not reach that of CTA, its ability to significantly improve the diagnostic sensitivity of aortic coarctation cannot be denied. Any methods or indicators that improve disease diagnosis should be given attention by echocardiography specialists.

This retrospective study demonstrated that abdominal aortic spectral Doppler combined with echocardiography improved diagnostic sensitivity for aortic coarctation in pediatric patients. ROC curve analysis identified clinically significant thresholds for abdominal aortic PSV, AT, and aortic isthmus *Z*-score, establishing valuable reference values for pediatric diagnosis and management. A standardized scanning process is crucial for accurate diagnosis. We should promote the application of abdominal aortic spectral Doppler ultrasound for the diagnosis of cardiovascular diseases in pediatric patients.

### Limitations

The limitations of this study include several factors. First, the sample size was insufficient, which may have introduced bias into the results, and abdominal aortic spectral Doppler was not analyzed in relation to intracardiac structural abnormalities. Second, only patients diagnosed with aortic coarctation through CTA were included, potentially excluding patients initially identified as possibly having aortic coarctation by echocardiography without subsequent confirmation by CTA, which could introduce selection bias. Third, due to the retrospective nature of the study, it was inherently subjected to the limitations of such research designs.

## Conclusion

Abdominal aortic spectral Doppler combined with echocardiography can increase the diagnostic sensitivity for aortic coarctation in pediatric patients. A tardus–parvus waveform of the abdominal aorta serves as an indirect indicator of aortic coarctation, guiding physicians to focus on specific regions when performing echocardiography and thereby increasing the precision of aortic coarctation diagnosis in pediatric patients.

## Data Availability

The original contributions presented in the study are included in the article/Supplementary Material; further inquiries can be directed to the corresponding authors.
